# Notes on Preparations for the Sick

**Published:** 1895-09

**Authors:** 


### Notes on Preparations for the Sick.

**Papaïn (Dr. Finkler & Co.): Powder; Pills; Lozenges; Tablets.**—B. KÜHN, London.—Much has been written on the Papaïn controversy, and on the whole this vegetable digestive from the Carica Papaya appears in by no means a disadvantageous light. It has been proved that it cannot be compared with Pepsine on account of its variety of digestive action, but this was taken as granted from the first. Its value as a digestive agent has, however, been proved to be in accordance with the experience of the natives who used the Papaw leaves.

Our own experiments have given the following results, showing that Papaïn is a more powerful digestive than the best of the animal pepsines in daily use, and is much less repulsive in character. The powder contains a slight amount of reducing sugar, a large quantity of albumen and also some albumose, but it has no action on starch. The action tested in acid, alkaline, and neutral media was as follows:—A mixture of stained fibrin and starch placed in test tubes with (1) .2 per cent. HCl; (2) $Na_2 CO_3$; (3) distilled water. Placed in warm chamber at 39° C. for one hour, then removed and tested. Action of the ferment takes place very readily in No. 2 tube, about ¼ as readily in No. 1 tube, and very slowly in No. 3 tube. This is evidenced by the depth of colour of solution which is formed by liberation of the carmine in the fibrin. On testing the solution they all contain varying quantities of *albumose*. They also contain very small quantities of peptone in following order: No. 2 most, No. 1 and No. 3 least.

---

**Diurnules: Arsenious Acid; Copper Arsenite; Nitro-Glycerine; Strophanthin; Hyoscyamine; Atropine Sulphate.**—Parke, Davis & Co., London.—**Diurnules** are intended to save the physician all doubt and trouble as to the dose of various toxic drugs. Twelve diurnules represent in equal subdivision the maximum internal dose *per diem* for an adult. When it is desirable to prescribe for an adult the full daily dose of any such active principle, one diurnule is to be administered every two hours. If the dose has to be reduced, the diurnule is given less often. There can be no possibility of inadvertently giving an overdose.

---

**Iron Jelloids.**—Warrick Brothers, London.—These are small flattened capsules, each of which contains the equivalent of ten grains of Pil. Blaud. They are made by mixing the freshly precipitated iron sub-carbonate with a jujube mass, by which the action of air on the iron salt is prevented. The light colour of the interior of these jelloids shows that the iron retains its unoxidised state. They are readily soluble in warm water, are easily swallowed as pills, and have little taste if leisurely masticated by those who cannot swallow a pill. They are a novelty, and an elegant form of a very favourite preparation of iron, on the original of which so many improvements have been successfully devised: these jelloids appear at present to be the climax of the series. Various sizes, and combinations with arsenic, have been prepared.

---

**Mist. Hepatica Conc.**—C. J. Hewlett & Sons, London.—Among other active constituents of this somewhat complex mixture are cascara, rhubarb, jalapin, podophyllin, and cocaine.

The combination has received a rather fanciful name indicating its especial action as a hepatic stimulant. A dose of one drachm has a decided aperient action and does not usually give griping or tenesmus. The components of the fluid are all useful drugs when acting independently of each other: they do not seem likely to be inharmonious when given together, and experience has shown that they assist each other in this unscientific combination.

---

**Somatose.**—Farbenfabriken vorm. Friedr. Bayer & Co., Elberfeld.—Peptones have not been altogether a success: they are always more or less nauseating, and their nutritive value has been much called in question. The Elberfeld firm say they have succeeded in their efforts to provide a preparation consisting almost entirely of albumoses, to which they have given the name **Somatose**, and for which Messrs. Bryce and Rumpff are the British agents. It is a light yellow powder, soluble in water, odourless and tasteless: it may be given by mouth or by hypodermic injection, it needs no digestion, and it contains no deleterious by-products: it must be rapidly absorbed, and should be invaluable in tiding patients through critical periods when the digestive powers are in abeyance. It may be given in solution in milk, coffee, cocoa, or gruel.

Our analysis shows that the powder contains a large quantity of peptone and dextrin, with slight amount of albumose and a trace only of kreatinin and of tyrosin.

---

**Anti-Diphtheritic Serum Exsiccatum.**—Burroughs, Wellcome & Co., London.—Each tube in which this preparation is sent out contains one gramme of the serum, prepared in the form of fine golden scales; it is freely soluble in twice its volume of cold water, and it represents ten cubic centimetres of the normal liquid. It will keep for any reasonable period, and is therefore always ready for use whenever a need for it may arise. Every care is taken to maintain the therapeutic activity of this preparation: each tube is marked with the date, the number of the horse, and the degree of immunity; these facts are certified by the medical director in charge of the Bacteriological Laboratory.

---

**Bronchi-fume.**—W. A. Manning, Birmingham.—This new remedy for asthma does not appear to differ much from the well-known powders in common use; but its agreeable aromatic odour, due to cubebs and aniseed, give it a distinctive character, and it of course contains the usual ingredients such as are found in all these powders (stramonium, cannabis indica, and nitrate of potash).

### MALE NURSES.

It must have been in the experience of most medical men that there is considerable difficulty in obtaining male nurses to attend on those who, from their peculiar ailments, rather resent the presence and manipulations of a woman. We have much pleasure in calling attention to the existence of an institution where not only male nurses can be obtained, but where the sobriety of the attendant is guaranteed, a by no means unimportant matter when dealing with the male sex. The Male Nurses (Temperance) Co-operation has been in existence for more than a year, and from the need there is for trustworthy sober men for nursing, we believe that only a more thorough knowledge that there is such an institution is required for its continued success. The secretary's address is 8 Great Marylebone Street, London, W.

